# β2-microglobulin is overexpressed in buccal cells of elderly and correlated with expression of p16 and inflammatory genes

**DOI:** 10.1016/j.sjbs.2022.103418

**Published:** 2022-08-19

**Authors:** Mohammad Althubiti

**Affiliations:** Biochemistry Department, Faculty of Medicine, Umm Al-Qura University, Makkah, Saudi Arabia

**Keywords:** β2M, Buccal cells, P16, Inflammatory genes, Aging

## Abstract

β2M (Beta 2 microglobulin) is a small protein that is found in all nucleated cells, previous finding showed that its levels increased in the serum of the elderly. Buccal cell samples are none invasive approach for assessing the expression of target genes. There was rationality to assess the expression of β2M in buccal cells of people of a different group of ages. Indeed, the expression of β2M increased significantly with fold change 3.40, 4.80, 6.60^**^, 8.20^***^ and 12.04^***^ for the group of age 18–25 years, 26–35 years, 36–45 years, 46–55 years, and 56–70 years respectively. The same observation was seen with markers of biological aging (p16^INK4a^) with fold change 3.19, 3.90, 4.80*, 8.50^***^ and 12.40^***^ for the group of age 18–25 years, 26–35 years, 36–45 years, 46–55 years, and 56–70 years respectively. As expected, there was an increase in the inflammatory genes (IL-1 β and IL-6) expression in the elderly. Moreover, there was a direct significant correlation (r = 90, p < 0.001) between β2M expression and age (years), and the same direct significant correlation between p16^INK4a^ expression and age (years) was also seen (r = 90, p < 0.001). In addition, a direct correlation between β2M and p16^INK4a^ was also seen (r = 0.8.3, p < 0.001), there was also direct correlation between β2M and IL-1 β and IL-6 with (r = 0.5, p < 0.001; r = 0.68, p < 0.001) respectively. This evidence showed that β2M increased in buccal cells of the elderly compared to younger, and thereby buccal cells can be exploited to assess biological aging by measuring β2M levels, however, large sample size and using another assessing method such as β2M protein levels should be performed to confirm the results.

## 1 Background

β2M is a small protein that is expressed in all nucleated cells, previous data showed that its activity increases during inflammation (Shi, 2009). β2M interplays with cytokines for instance, IL-6, IL-8 and others intracellularly to induce inflammatory responses. In addition, it can bind and modulate the activity of growth factors and hormones and receptors (Balint, 2001, Yang, 2007). As a cancer promoting factor, it has been shown to be a growth factors and has been associated with cancer formation(Huang, 2006). β2M high levels stimulate stem cells via promoting IL-6 activity resulting in cancer cells invasion and metastasis (Zhu, 2009). On another context, it has been shown to have an apoptotic role in many liquid cancers (Mori, 2001, Gordon et al., 2003). β2M is accumulated in joints of patients are under renal dialysis for long term (Portales-Castillo et al., 2020), genetic mutation in β2M is associated with amyloid fibrils (Leney et al., 2014).

β2M has been exploited as a biomarker for many disorders (Banner, 1990, Mogi, 1995, Mogi, 1994, Gao et al., 1994, Kim et al., 2014, You et al., 2017, Argyropoulos, 2017, Wang, 2018, Saddiwal et al., 2017, Behairy, 2017). There are several studies that link β2M levels in serum and presence of cancers. In multiple myeloma (MM), β2M is used for prognostic purposes (D'Anastasi, 2014, Molica, 1999). In addition, β2M can also be measured in cerebrospinal fluid (CSF) to assess nervous system diseases (Jeffery et al., 1990). β2M has been shown to be high in serum of patients with late-stage prostate cancers. Increasing of β2M levels in these patients might be because of positive effect of androgen that stimulates β2M secretion (Mink et al., 2010).

In addition to the previous, in inflammatory bowel diseases, β2M can be used a diagnostic biomarker (Yilmaz, 2014). In infectious diseases such as cytomegalovirus and human immunodeficiency virus infections, β2M has been shown to elevated (Chitra et al., 2011), which could explain its role in immune system. In bacterial infection such as Helicobacter pylori infection, β2M level is elevated in gastric biopsies that again underscores infiltration of inflammatory cells (Conz et al., 1992). In organ donation, β2M has been used for monitoring for successful renal transplantation (Astor et al., 2013). These evidences are examples of using of β2M as a biomarkers in clinical settings of many diseases. Previous study by our group showed that β2M expressed highly in senescent cells (old cells) (Althubiti et al., 2014), recently, it has been shown by our group that β2M expressed highly in blood samples of old people comparing to youngers. Furthermore, we have shown that β2M correlated significantly with oxidative stress biomarkers, which could underscore a potential role in oxidative stress network (Althubiti, 2021). Therefore, there is a rationality to test the expression of β2M across different group of age using other easier source of sample such as buccal cells.

Buccal cells are epithelial cells that is similar to brain and skin in nature. They are originated from ectodermal differentiation during embryonic development. Buccal cells can be collected easily by different method that described previously(Richards, 1993, King, 2002, Hayney et al., 1996, Feigelson, 2001). These methods are showed high number of cells that can be used for different biological assays (Hattori, 2002, Spivack et al., 2004). Comparing to other sample methods, buccal cell samples are less invasive and very easy to collect. In addition, buccal cells are very stable after isolation from mouth(Lee et al., 1994), which makes them easy to process and analyze. Moreover, buccal cells are easy to preserve in buffer (François et al., 2014). These make them an easy source for diagnosis. In this study, we used buccal cells to examine the expression of β2M in different age groups. In addition, we correlated the expression of β2M with p16^INK4a^ a biological biomarker of aging(Liu et al., 2009, Ressler et al., 2006, Zuev et al., 2019).

## 2 Methods

### 2.1 The study subjects

A cross-sectional study design was used to gather data from 81 participants, who were then divided into five age groups. In the first group, participants were aged 18 to 25, in the second, 26 to 35, in the third, 36 to 45, in the fourth, 46 to 55, and in the fifth, 56 to 70. HAPO-02-K-012–2021-03–600 is the approval number for the Medical Ethics Committee of the Faculty of Medicine at Umm Al-Qura University, which approved the sampling procedures in accordance with the Declaration of Helsinki of 1975 and the written informed consent of all participants, as well as nutritional and habitat data collected in accordance with the study form item (Table 2). All subjects appear to be in good health and free of any long-term conditions.

### 2.2 Sampling

To preserve RNA during buccal cells collection, iSWAB RNA v2 Collection Kit (mawi, Biosamplin Reinvented) was used to collect buccal cells according to the manufacture’s protocol. For preparing iSWAB samples to be used directly in RT-PCR, collected buccal samples with iSWAB-RNA were incubated>3 hrs at room temperature before processing in RT-PCR. The samples were then centrifuged the for 2 min at 14000 rpm then 1uL of the clarified supernatants were taken and diluted in nuclease-free water to 1:16, finally taken 2 uL from the iSWAB diluted sample and applied direct on RT-PCR.

### 2.3 The primer designs

Primers were ordered from (Integrated DNA Technologies). NCBI's BLAST database was used to generate a primer for an individual gene by using the “BLAST” function (https://blast.ncbi.nlm.nih.gov/blast.cgi). With amplicons shorter than 200 base pairs, primers were created (bp) (Table 1). Melting curve analysis confirmed the primer sets' specificity in this study. Endogenous GAPDH was used to maintain a constant level of gene expression during the study.

**Table 1:** Details of the primer sequences of studied genes.

	NM	Gene ID	Forward Sequence	Reverse Sequence
beta 2 Microglobulin (B2M)	Human qPCR Primer Pair (NM_004048)	567	CCACTGAAAAAGATGAGTATGCCT B2M F: AGCAGAGAATGGAAAGTCAAA	CCAATCCAAATGCGGCATCTTCA B2M R: TGTTGATGTTGGATAAGAGAA
IL1 beta (IL1B)	Human qPCR Primer Pair (NM_000576)	3553	CCACAGACCTTCCAGGAGAATG iL: ACAGGATATGGAGCAACAAGTGG	GTGCAGTTCAGTGATCGTACAGG IL: GGGCTTATCATCTTTCAACACGC
IL6	Human qPCR Primer Pair (NM_000600)	3569	AGACAGCCACTCACCTCTTCAG: GGGCTTATCATCTTTCAACACGC	TTCTGCCAGTGCCTCTTTGCTG ATTTTCACCAGGCAAGTCTCCTC
p16	Human qPCR Primer Pair (NM_058195)	1029	CTCGTGCTGATGCTACTGAGGA	GGTCGGCGCAGTTGGGCTCC

**Table 2:** The demographical and descriptive statistical data of the studied subjects.

		G1	G2	G3	G4	G5
Number		21	17	11	18	14
Age (Years)	Mean ± SD	22 ± 2.8	30.4 ± 3.2^a^	41.7 ± 3.35^a^	50.7 ± 2.9^a^	64.7 ± 5.8^a^
	Range	18–25	26–35	37–45	46–55	56–70
BMI (Kg/m^2^)	Mean ± SD	20.5 ± 3.2	22.1 ± 2.7	21.4 ± 2.1	22.2 ± 2.6^c^	23.8 ± 1.8 ^a^
	Range	17.5–30	18–26	18–24	18–25	19–26
Water drinking (L)	Mean ± SD	3.5 ± 0.5	2.8 ± 0.9	3 ± 0.8	2.8 ± 0.4	3.2 ± 0.4
	Range	1.5–5	1–5	2–4	2–3	3–4
Smoking	Non-smoker	15 (71.4 %)	8 (47.1 %)	6 (54.5 %)	12 (66.7 %)	12 (85.7 %)
	Smoker	6 (28.6 %)	9 (52.9 %)	5(45.5 %)	6 (33.3 %)	2 (14.3 %)
Exercise	No exercise	19 (90.5 %)	9 (52.9 %)	6 (54.5 %)	14 (77.8 %)	9 (64.3 %)
	Walking	2 (9.5 %)	3 (17.6 %)	2 (18.2 %)	4 (22.2 %)	5 (35.7 %)
	Cycling	–	4 (23.5 %)	1 (9.1 %)	–	–
	Football	–	1 (5.9 %)	2 (18.2 %)	–	–
Supplemental Intake	No-supplements	21 (100 %)	14 (82.4 %)	10 (90.9 %)	18 (100 %)	10 (71.4 %)
	Multi-vitamins	–	2 (11.8 %)	1 (9.1 %)	–	5 (28.6 %)
	Protein	–	1 (5.9 %)	–	–	–
Vegetable Eating	No	7 (33.3 %)	7 (41.2 %)	2 (18.2 %)	11 (61.1 %)	1 (7.1 %)
	Sometimes	11 (52.4 %)	8 (47.1 %)	8 (72.7 %)	3 (16.7 %)	4 (8.6 %)
	Daily	3 (14.3 %)	2 (11.8 %)	1 (9.1 %)	4 (22.2 %)	9 (64.3 %)
Sleeping hours	8H	9 (%)	4 (23.5 %)	1 (%)	10 (55.6 %)	9 (64.3 %)
	< 8H	11 (52.4 %)	10 (58.8 %)	9 (81.8 %)	6 (33.3 %)	5 (35.7 %)
	> 8H	1 (4.8 %)	3 (17.6 %)	1 (9.1 %)	2 (11.1 %)	–

### 2.4 Extraction of RNA and synthesis of complementary DNA (cDNA)

The manufacturer's RNA isolation kit was used to obtain total RNA from the buccal cells (Invitrogen; Thermo Fisher Scientific, Inc., USA). A Genova Nano Micro-volume, Life Science & Standard Spectrophotometer was used to determine the concentration of RNA in the sample. The Veriti Thermal Cycler System (Applied Biosystems®, Thermo Fisher Scientific, Inc., USA) was used in accordance with the manufacturer's instructions to reverse-transcribe 500 ng of RNA into cDNA (Takara Bio, Inc.). The cDNA was synthesized from total RNA as previously described (Althubiti et al., 2020).

### 2.5 RTPCR

The Fast RT-PCR 7500 System was used in accordance with the manufacturer's instructions to perform qPCR using the Applied Biosystems^TM^ SYBRTM Green master mix (Thermo Fisher Scientific, Inc., USA). SYBR^TM^ Green master mix was added to 4 μl of the diluted cDNA template, and 500 nM of each primer was mixed in equally. This is a list of the 40-cycle PCR standards. For each run, a baseline and a threshold were assigned automatically (7500 Fast Software, Version 2.0.5). Cycle threshold (Ct) is established when fluorescence exceeds a predetermined threshold (Ct). The 2^-ΔΔCt^ method was used to compare the relative levels of expression of various genes in this study.

### 2.6 Statistical analysis

The mean ± standard deviation is used to present data. The analysis of variance (One-Way ANOVA) statistical method was applied to compare groups with assumed that the sample is drawn from the normally distributed population and the population variance is equal. Based on variance equality, the One-way ANOVA test with Tukey’s HSD post-hoc tests were used to compare between the groups. Correlation coefficients between individuals (r value) were used to assess the genetic correlation with age. The P value was considered statistically significant if it was<0.05 in all statistical analyses (GraphPad Software Inc.).

## 3 Results

Eighty-one subjects were recruited in the study and divided them into five age groups as previously described in the method. Different demographical and descriptive information were documented from the participants such as age, BMI, quantity of water drinking, smoking status, exercise and supplement & vitamins intake as shown in Table 1.

### 3.1 β2M expression increased in buccal cells of elderly

**Fig. 1:**
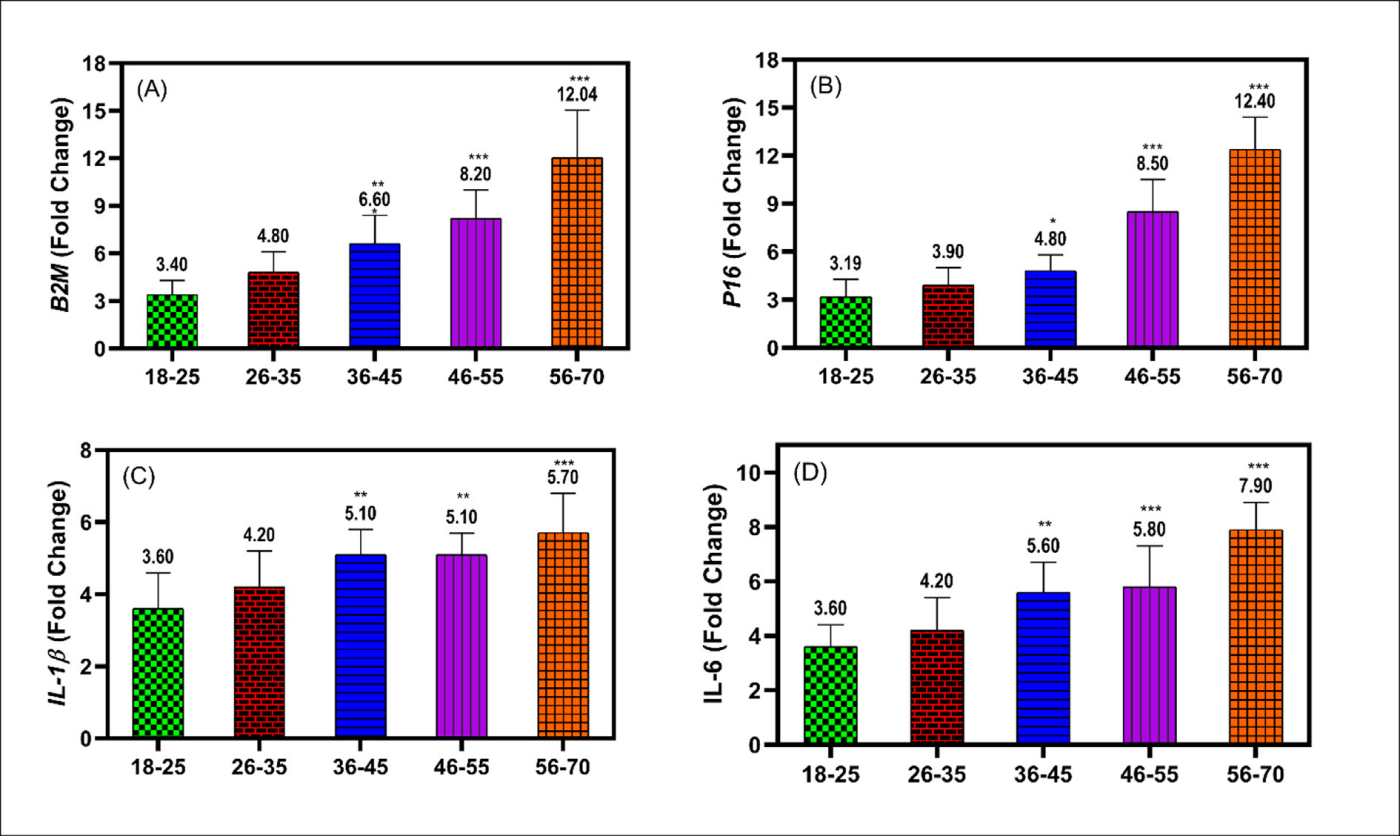
The fold change of the mRNA expression levels of studied genes B2M (A), P16^INK4^ (B), IL-1β (C), and IL-6 (D). The data expressed as mean ± SD of the fold change. *, **, and *** indicate the P values < 0.05, P < 0.01, and P < 0.001 of the significant difference levels between the age groups and the younger group (>25 years) as a control, respectively.

In the study, we examined the expression of β2M in different age groups, as depicted in the Fig. 1 panel A. Indeed, there was significant differences of β2M expression in the elderly groups comparing to the younger group, as shown in the Fig. 1. In the group of (36–45 years) there was a significant increase in the fold change of β2M (6.60) comparing to the younger group (18–25 years) (3.40) p value < 0.01^**^. In addition, older group of age (46–55 years) showed more significant difference in the β2M expression (fold change 8.20) comparing to the younger group (18–25 years) p value < 0.001^***^. The same significant difference was also seen also in the older group of age (56–70 years) (fold change 12.04) comparing to the younger group of age p value < 0.001^***^, as shown in the Fig. 1panel A. To assess the biological aging of the participants, p16 ^INK4^ expression was used, as shown in the Fig. 1 panel B. The expression of p16 ^INK4^ showed a significant difference in the fold change (4.80) in the group (36–45 years) comparing to the fold change (3.19) in the younger group (18–25 years) p value*<0.05. The expression of p16 ^INK4^ was seen in the group of age (46–55 years) fold change (8.50) p value^***^ < 0.001 comparing to the younger group of age (18–25 years). The same significant difference was seen in the older group of age (56–70 years) (12.40) fold change comparing to the younger group of age (18–25 years) p value^***^ < 0.001, as shown in the Fig. 1 panel B.

After assessing the mRNA expression of β2M and p16 ^INK4^ in different group of age, we sought to measure the expression of inflammatory biomarkers in these groups, as shown in the Fig. 1 panel C & D. Study participants aged 36 to 45 years showed a significant increase of fold change of IL-1 expression (5.10) compared to the younger group (3.60 fold change) p value** < 0.01. In addition, a significant increase also was seen in in the group age (36–45 years) with fold change of IL-1β expression (5.10) comparing to the younger group of age (3.60 fold change) p value^**^ < 0.01. A more significant difference was seen in the older group of age (56–70 years) with 5.70 fold change comparing to the younger group of age p value^***^. < 0.001. No significant difference was seen in the fold difference between 26 and 35 years and 18–25 years, as shown in the Fig. 1 panel C.

Moreover, mRNA expression of IL-6 was also investigated. A significant fold difference was seen in the group of age (36–45 years) (5.60 fold change) comparing to the younger group of age (18–25 years) that was only 3.60 fold change, p value^**^ < 0.01. A more significant difference in the fold change was noticed in the (46–55 years) (5.80 fold change) comparing to the younger group of age (18–25 years), p value^***^ < 0.001. Same significant difference was also seen in the older group of age (56–70 years) with fold change 7.90 comparing to the younger group of age (18–25 years) with p value^***^ < 0.001, as shown in the Fig. 1 panel D.

### 3.2 β2M expression in buccal cells correlated directly with age

**Fig. 2:**
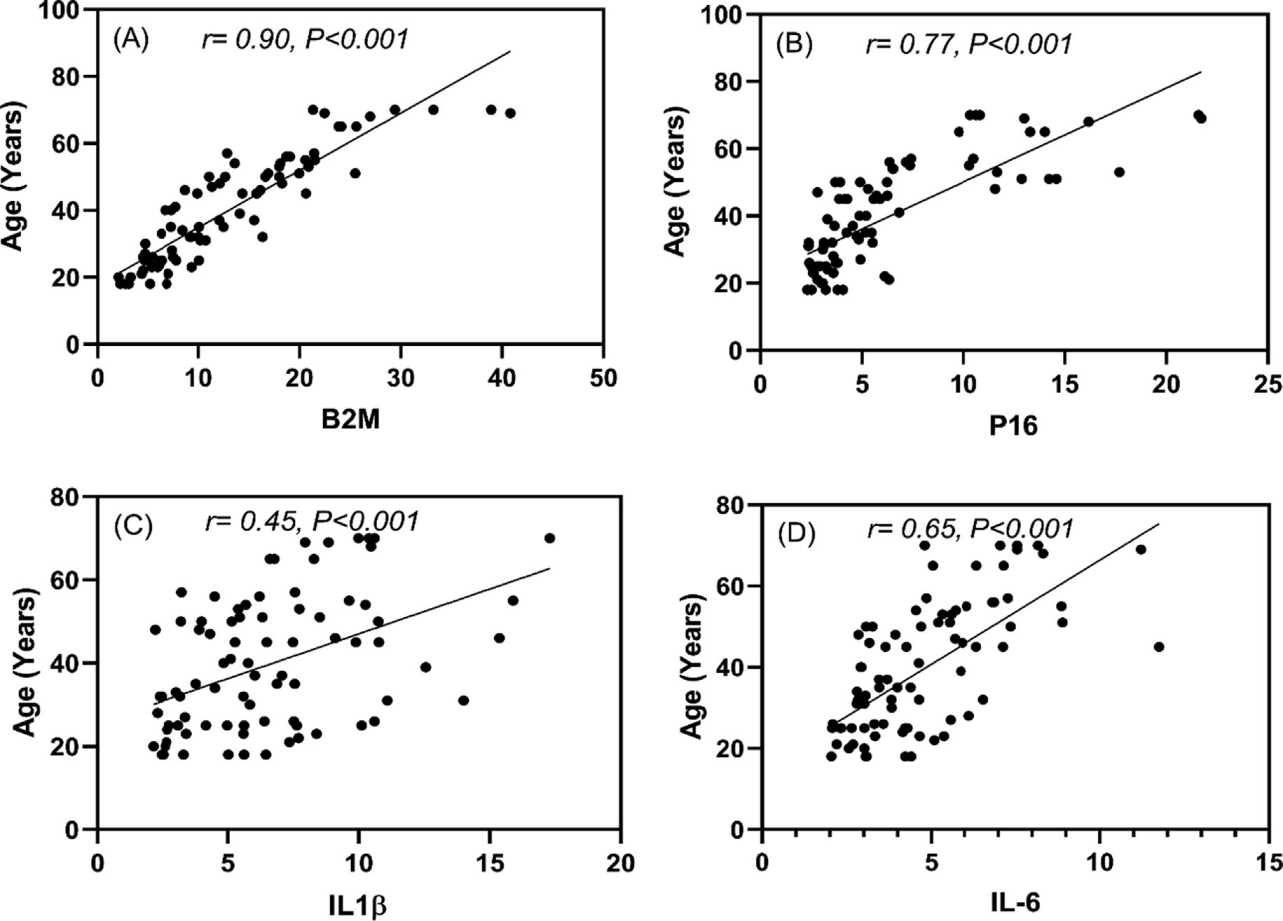
The Pearson correlation coefficient of studied genes and subjects' age. Each set of age and/or gene expression data has a significant positive linear correlation (r-value, P < 0.001). It is the product of two variables' covariances and thus a normalized measurement of covariance.

After studying the expression of β2M, p16^INK4^ and the inflammatory genes in different group of genes, we sought to assess the correlation between β2M, p16^INK4^, the participants' inflammatory biomarkers and age. Fig. 2A shows a strong direct correlation between 2 M and participant age (r = 0.90, p = 0.001). A direct correlation between the biomarker of biological aging, p16^INK4^, and age of participants was also observed (r value = 0.77, p value < 0.001) as shown in the Fig. 2B. In addition, inflammatory genes IL-1β & IL-6 showed moderate direct correlation with age with r value = 0.45, p value < 0.001 r value = 0.65 and p value < 0.001 respectively, as shown in the Fig. 2C&D.

**Fig. 3:**
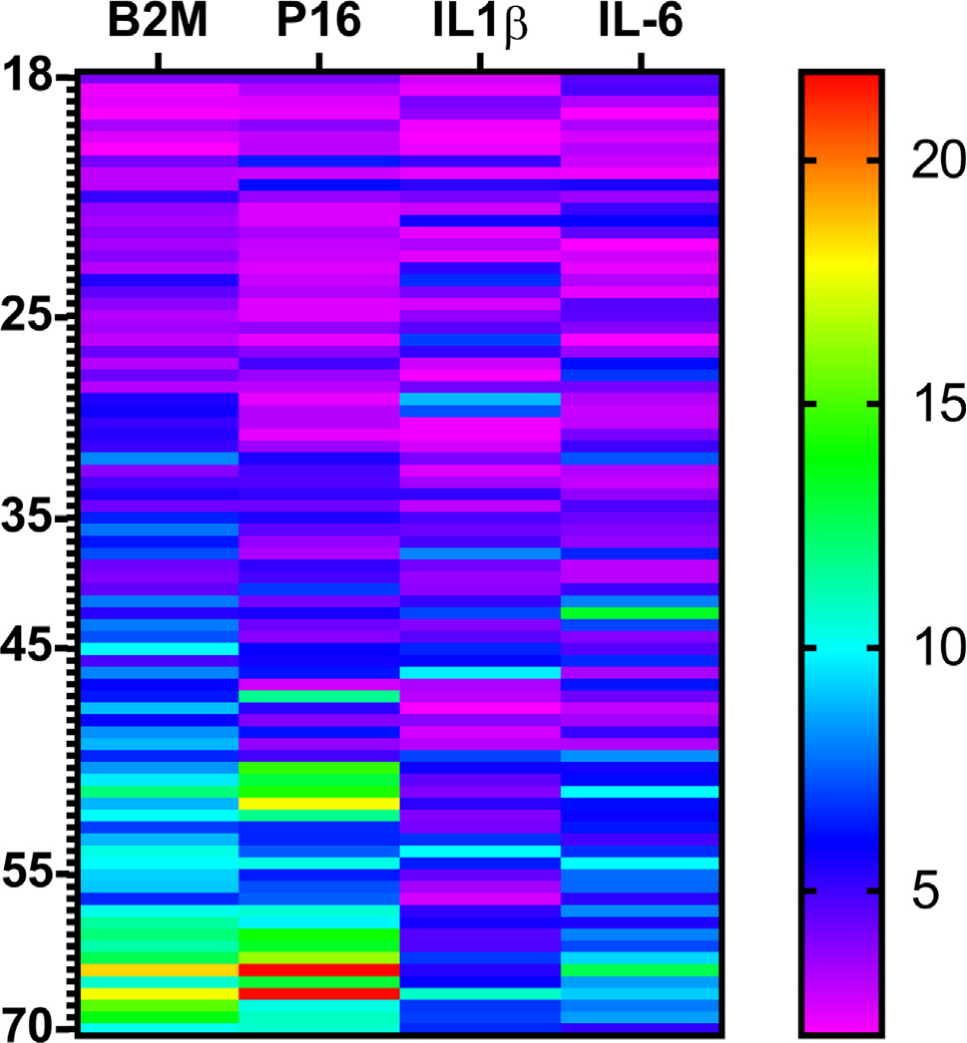
The Expression levels heatmap with cluster analysis. The colour intensity in each box shows the percentage of expression levels (Fold change) for each gene relative to the colour key on the right side in correspondence to the participants age in the left side. The subject sample notation in the left side. (There were significant differences between P16 “as a common aging gene” and both IL-1b (p < 0.01) and IL-6 (p < 0.05) genes, however non-significant differences were observed between P16 and B2MG (p < 0.57).

**Fig. 4:**
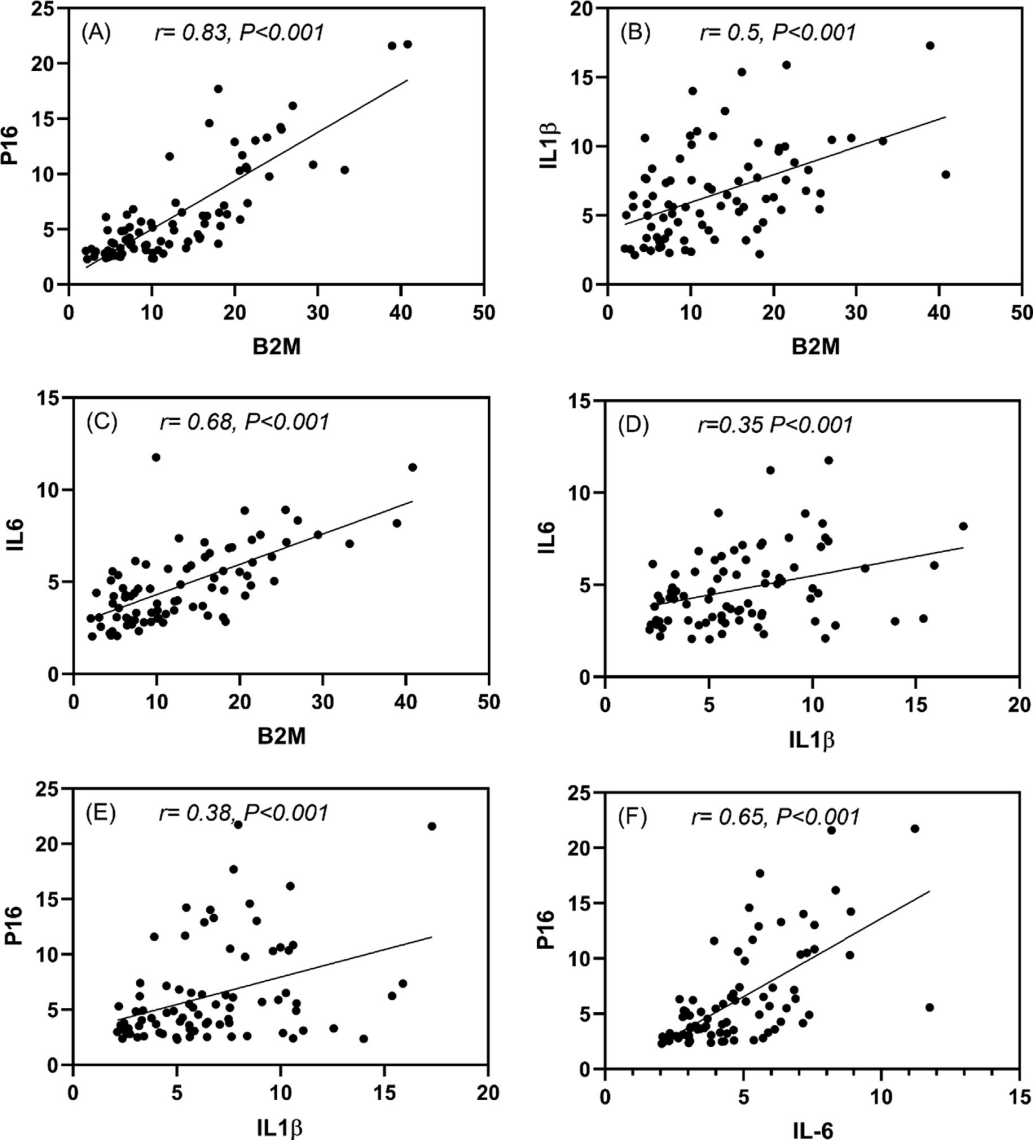
The Pearson correlation coefficient of studied genes. Each set of gene expression data has a significant positive linear correlation with correspondence gene (r-value, P < 0.001). It is the product of two variables' covariances and thus a normalized measurement of covariance.

To deep illustrate the relationship between studied genes and age, color intensity of the percentage genes expression levels (Fold change) was created for each sample in relation to the age and was presented in the form of heatmap with cluster analysis as shown in the Fig. 3. Elderly participants showed high percentage of β2M expression levels (Fold change) comparing to the youngers as shown in the Fig. 4. In addition, elderly people also showed high percentage of p16^INK4^ expression levels (Fold change) comparing to the youngers. Less intensity of IL-1b and IL-6 expression levels (Fold change) were seen in elderly people comparing to β2M and as shown in the Fig. 3. p16^INK4^ and IL-1b (p < 0.01) and IL-6 (p < 0.05) genes showed significant differences, but p16^INK4^ and β2M (p < 0.57) showed no significant differences.

### 3.3 β2M expression correlated directly with expression of p16^INK4^ in buccal cells

When we looked at the gene expression of p16INK4 β2M, p16INK4 and the genes of the inflammatory in different age groups, we found a strong correlation. β2M expression showed a significant direct correlation with p16^INK4^ expression (r value = 0.83, p value < 0.001), as shown in the Fig. 3A. In addition, β2M expression also showed a significant direct correlation with inflammatory genes IL-1β & IL-6 expressions with r value = 0.5, p value < 0.001 and r value = 0.68 and p value < 0.001 respectively, as shown in the Fig. 3B&C. The same correlation was seen between p16^INK4^ expression and inflammatory genes IL-1β & IL-6 expressions with r value = 0.38, p value < 0.001 and r value = 0.65 and p value < 0.001 respectively, as shown in the Fig. 3. There was significant correlation between studied genes especially in the older group of age G4 and G5 groups, as shown in the supplementary table1.

The Pearson correlation coefficient of studied genes and subjects' age groups. Each set of age and/or gene expression data has a significant positive linear correlation (r-value, ***P < 0.001, **P < 0.01, and * P < 0.05). It is the product of two variables' covariances and thus a normalized measurement of covariance.

## 4 Discussion

β2M is a small peptide that plays roles in inflammation and immunity. It has been documented that β2M can be used in diagnosis and/ or monitoring some of infectious and malignant diseases(Shi, 2009, Portales-Castillo et al., 2020, Banner, 1990, Svatoňová, 2014, Toth et al., 2013). previous work from our group showed that its blood levels is high in elderly(Althubiti, 2021). This work revealed that β2M levels were also high in buccal cells of old people. There was also a high correlation between β2M expression and p16^INK4^ expression, as well as inflammatory gene expression.

p16^INK4^ expressions were found to be high in blood T cells of old people(Liu et al., 2009), in skin ageing (Ressler et al., 2006) and in buccal cells of elderly people (Zuev et al., 2019). These showed that p16^INK4^ is a biomarker of aging in the studied samples or tissues. In this study p16^INK4^ has been used as a control for β2M expression, β2M expression is higher and more significant than the expression of p16^INK4^ in the age group (36–45 years) comparing to the younger group of age, with same significant increase for both genes in older groups. Using β2M as a biomarker of biological ageing even in middle-aged people was supported by this study. In addition, the correlation between the β2M and age showed that a strong direct correlation even more than the correlation between p16^INK4^ and age. Previously, β2M has been shown to be highly expressed in the plasma membrane of senescent cells(Althubiti et al., 2014), later work by our group validated the potential use of β2M as a biomarker of aging in blood (Althubiti, 2021). Here different sampling method was used to assess the β2M levels, which provide a simple and non-invasive approach. This also underlined the potential ability of β2M to be used as a biomarker for aging.

Increasing the expression of β2M in buccal cells of elderly people has advantages and implications. Firstly, the source of sample is not invasive comparing to other biological sources. In addition, in the future, any assessment of the biological aging and inflammation and / or chronic diseases can be evaluated in non-invasive way. Moreover, this method can be commercialized easily. However, further study should be conducted to include larger sample size of smoker and non-smoker participants to reveal the effect such factor on the expression of β2M.

The data showed that β2M correlated directedly and more significantly with inflammatory genes expressions comparing to the p16^INK4^ expression. This suggested that β2M could have a role in inflammation in elderly. Indeed, β2M have been shown to increase in inflammation and / or inflammatory associated disorders (Yılmaz, 2014, Topçiu-Shufta, 2016, Xie and Yi, 2003). Our previous work also showed the association between oxidative stress and β2M expression (Althubiti, 2021). The exact role and relationship between β2M and the inflammatory and oxidative stress cascade needs to be clarified, however, in future studies.

The study has limitations of being small sample size, therefore larger sample size should be included to confirm the findings. In addition, future work should also assess protein levels of β2M and compare it to the mRNA expression to verify the findings.

In summary, the results showed that β2M expressed highly in buccal cells in comparisons between the elderly and younger people. β2M has a significant direct correlation with age of participants. Moreover, its expression directedly correlated with inflammatory biomarkers, these suggested the potential use of β2M as biomarker of biological aging. However, future work should include larger sample size before translating the findings for commercial or diagnostic purposes.

## Declaration of Competing Interest

The authors declare that they have no known competing financial interests or personal relationships that could have appeared to influence the work reported in this paper.
