# Supplementary material for: β2-microglobulin is overexpressed in buccal cells of elderly and correlated with expression of p16 and inflammatory genes
Source: Saudi J Biol Sci. 2022 Aug 19;29(10):103418. doi: 10.1016/j.sjbs.2022.103418 (PMC9440304; doi:10.1016/j.sjbs.2022.103418)
Supplement: Supplementary data 1 [file mmc1.docx]

**Table 1S:**

**The Pearson correlation coefficient of studied genes and subjects' age groups. Each set of age and/or gene expression data has a significant positive linear correlation (r-value, ***P<0.001, **P<0.01, and * P<0.05). It is the product of two variables' covariances and thus a normalized measurement of covariance.**

|  |  | B2M | p16 | IL-1B | IL-6 |
| --- | --- | --- | --- | --- | --- |
| B2M | G1 | 1.00 | 0.20 | 0.40 | 0.05 |
|  | G2 | 1.00 | 0.32 | 0.12 | 0.29 |
|  | G3 | 1.00 | -0.29 | 0.37 | 0.07 |
|  | G4 | 1.00 | 0.51* | 0.11 | 0.34 |
|  | G5 | 1.00 | 0.83*** | 0.71*** | 0.74*** |
| P16 | G1 | 0.20 | 1.00 | 0.24 | 0.17 |
|  | G2 | 0.32 | 1.00 | -0.30 | 0.43 |
|  | G3 | -0.29 | 1.00 | -0.20 | 0.10 |
|  | G4 | 0.51* | 1.00 | 0.02 | 0.37 |
|  | G5 | 0.83*** | 1.00 | 0.63** | 0.73*** |
| IL-1B | G1 | 0.40 | 0.24 | 1.00 | 0.08 |
|  | G2 | 0.12 | -0.30 | 1.00 | -0.46 |
|  | G3 | 0.37 | -0.20 | 1.00 | 0.54 |
|  | G4 | 0.11 | 0.02 | 1.00 | 0.22 |
|  | G5 | 0.71*** | 0.63** | 1.00 | 0.31 |
| IL-6 | G1 | 0.05 | 0.17 | 0.08 | 1.00 |
|  | G2 | 0.29 | 0.43 | -0.46 | 1.00 |
|  | G3 | 0.07 | 0.10 | 0.54 | 1.00 |
|  | G4 | 0.34 | 0.37 | 0.22 | 1.00 |
|  | G5 | 0.74*** | 0.73*** | 0.31 | 1.00 |
